# An Experience with the New Anæsthetic—Chloroform, Ether and Absolute Alcohol

**Published:** 1883-02

**Authors:** 


### AN EXPERIENCE WITH THE NEW ANÆSTHETIC—CHLOROFORM, ETHER AND ABSOLUTE ALCOHOL.

A young lady aged eighteen, whose lower teeth were so carious that only two out of the complete set were considered good enough to retain (the result of hereditary syphilis), was put under the influence of the above anæsthetic by her regular medical attendant, who has had great experience in its use, to give me the opportunity of excising the six incisors, extirpating the nerves and extracting the rest. The means adopted to administer it were very simple, the whole apparatus consisting of a thick felt cone five inches long, covered with oil-silk, with an opening to go over the mouth and nose and a hole at the apex to admit the air. Two drachms of the mixture were poured over the felt and applied gently to the face, and in two minutes and a half anæsthesia was complete. I excised the two incisors and drilled into the pulp-cavities, having fixed the mouth open with a Coleman's gag. The administration was continued, and I extirpated the nerves.

As there was every symptom of sickness, I did not proceed farther, and about half a pint of cream-colored vomit was thrown up. In about two minutes all was ready to proceed, so I extracted the teeth and made preparations for the restoration of consciousness. The pulse was quite steady and the hands and feet particularly warm, the respiration was regular, but no sign of sensibility presented itself. The face was slapped with a damp napkin and some liq. ammon. fort. applied to the nostrils. Five minutes elapsed and the nitrate of amyl was given in the usual way, with the effect of increasing the pulse and bringing a little more color to the face. Time went on and consciousness was as far off as ever.

A teaspoonful of brandy was put into the mouth, which induced vomiting. The ammonia was again applied to the nostrils with a slight sign of discomfort, as the head moved a little away from it. Having read that if mice were put under the influence of chloroform and were held up by their tails they would make attempts to bite, inversion was tried by tilting the operating-chair back until the head-rest touched the ground, without the least beneficial effect. Thirty minutes having elapsed and, beyond the prolonged insensibility, no unfavorable symtom presenting itself, we came to the conclusion that the patient was under the influence of the alcohol. By repeating the slapping and ammonia, which was now decidedly objected to, consciousness returned, and in forty minutes after the operation ceased she was walking about the room with a most unsteady gait, and complained of feeling dreadfully sleepy.

We induced her to have her cloak put on, and in a few minutes she left in her brougham with her friends.

I saw her three days after, when she said she did not suffer during the remainder of the day more than an attack of sickness, when she brought up a quantity of blood, which had evidently been swallowed, and a slight sense of drowsiness. The prolonged insensibility is quite unusual, but the happy freedom from the sickness which invariably follows the administration of chloroform is the rule, and in that, I think, favors the use of this anæsthetic.—*Henri Weiss, in British Journal of Dental Science.*

*Remarks.*—English surgeons are quite extensively using this mixture of chloroform, ether and alcohol as an anæsthetic, for what good reason we must confess our inability to perceive. Chloroform and ether in their administration are each accompanied by a separate train of symptoms, and the effect of each upon some of the vital organs is quite distinct. Each is fraught with special danger to patients laboring under certain diatheses, but neither can act as an antidote to the other. Their combination, therefore, multiplies the chances for a fatal termination, without offering any compensating advantages in convenience to the operator. Even were this latter the case, it could not properly be taken into consideration. When an operation hazardous to human life is to be performed, there should be but one question asked—what is the method involving least risk to the patient? The mere convenienee of the operator, the chance of any trivial nausea, the saving of a few moments of time, are not factors in the case.

If there be no heart lesions, no alcoholism and no state of extreme nervous irritability, chloroform vapor, properly diluted, may be as safe an anæsthetic as any, but its administration is, in the earlier stages, usually accompanied by hyperæsthesia and great excitement, due to the stimulating effects of the agent upon the sympathetic nerve system, and if the heart be weakened by disease its spasmodic struggles may result in paralysis of that organ.

Ether narcosis is seldom attended with the violent excitement stage common in chloroform, and does not so seriously depress the heart in its later action, but its administration may result in arrest of respiration, due either to a paralysis of the sympathetic nerves, or more frequently to the filling up of the smaller bronchi by mucous, through the destruction of sensibility in the membrane lining them and the consequent cessation of the ciliary motion, which should carry the mucous forward until expectoration can remove it.

The principal source of danger in chloroform narcosis, then, is from stoppage of the heart; in ether from suspension of respiration. We have performed very many experiments in anæsthesia upon the lower animals, and have demonstrated to hundreds of dentists and physicians the constant fact that in death from chloroform the heart first fails in its action, while in death from ether it continues its movements for a greater or less time after respiration ceases. In some hundreds of experiments with ether upon the smaller animals, such as dogs, cats, rabbits, etc., no life was ever lost unless purposely sacrificed. Artificial respiration, if resorted to in time, invariably restored them, because the heart was yet in action and circulation was unimpaired. The reverse of this is true in the use of chloroform, and hence an animal cannot always be revived in case of unfavorable symptoms.

But sulphuric ether has no power to stimulate a depressed or paralyzed heart, nor is chloroform of any use in cases of suspended respiration. What good reason is there, then, for administering a mixture of the two? Does not the operator necessarily double the danger by subjecting his patient to the hazards of both?

The addition of alcohol dilutes the anæsthetic mixture, and thereby diminishes the danger of giving an atmosphere too heavily charged with the vapor. It also acts as a stimulant, and thus assists in the diffusion of the agent as well as in its final elimination. But if it be this latter effect which is desired, a much better way is to administer it separately.

We submit that the administration of a mixture of anæsthetic agents unnecessarily complicates the operation; that it is unscientific, and is attended by an increased risk to the life of the patient.—[Ed., W. C. B.]

